# Role of Ultrasound-Based Therapies in Cardiovascular Diseases

**DOI:** 10.1016/j.shj.2024.100349

**Published:** 2024-07-16

**Authors:** Amit Bansal, Bernard Bulwer, Ricarda von Krüchten, Jagkirat Singh, Rajan Rehan, Ata Doost

**Affiliations:** a Bergen COVID-19 Research group and Influenza Centre, Department of Clinical Science, University of Bergen, Bergen, Norway; b Department of Infectious Diseases, University of Melbourne, at the Peter Doherty Institute for Infection and Immunity, Melbourne, Australia; c Cardiovascular Imaging Core Laboratory, Cardiovascular Division, Department of Medicine, Brigham and Women’s Hospital, Harvard Medical School, Boston; d Stanford University, School of Medicine, Stanford, United States; e University of Freiburg, Medical Center, Freiburg, Germany; f Department of Neurology, Creighton University, School of Medicine, Omaha; g University of Sydney Medical School, Sydney, Australia; h Macquarie University, Faculty of Medicine and Health Sciences and Macquarie University Hospital, Sydney, Australia

**Keywords:** Cardiovascular disease, ECSWT, Low-intensity ultrasound, HIFU, Extracorporeal cardiac shock wave therapy, High-intensity focused ultrasound, LIU, Therapeutic ultrasound

## Abstract

Cardiovascular diseases (CVDs) remain the leading cause of morbidity and mortality globally, placing an immense burden on health care costs worldwide. The emergence of therapeutic ultrasound-based therapies in the CVD management represents a promising innovative strategy beyond current established approaches. This paper explores three distinct modalities of ultrasound-based therapies—high-intensity focused ultrasound, extracorporeal shock wave therapy, and low-intensity pulsed ultrasound—each characterized by unique acoustic parameters and mechanisms of action tailored to specific therapeutic outcomes. High-intensity focused ultrasound was shown to be beneficial as an adjunct in the treatment of myocardial infarction and arrhythmias. It has also been investigated for the *in vivo* treatment of resistant hypertension, symptomatic aortic valve stenosis, arterial stenosis, tumors, hypertrophic cardiomyopathy, and external cardiac pacing. Extracorporeal shock wave therapy was shown to be beneficial in the treatment of chronic refractory angina pectoris, while low-intensity pulsed ultrasound was shown to be beneficial in dissolving blood clots and improving blood flow in the treatment of acute pulmonary embolism, despite its association with an increased risk of bleeding. Ultrasound-based therapies are, therefore, a potential adjunct and comparatively safe adjuncts for managing challenging CVD cases. Further investigations are essential to validate their long-term effectiveness and safety, particularly for high-risk individuals susceptible to postprocedural complications.

## Highlights

• Ultrasound-based therapies emerge as an add-on treatment for cardiovascular diseases.
• Indications: myocardial infarction, arrhythmias, hypertension, valve stenosis, clot lysis.
• Tolerable adjunct option for challenging cardiovascular diseases.
• Needs validation in high-risk patients (protocols and long-term effects).

## Introduction

Cardiovascular diseases (CVDs) continue to be the world's leading causes of morbidity and mortality, with a major impact on health care expenditure.^1^^,^^2^ Diagnostic medical ultrasound with frequencies in the megahertz range (MHz), including Doppler ultrasonography, has established clinical utility in the diagnosis of cardiovascular structural and functional abnormalities, including intracardiac and blood flow hemodynamics within the heart and blood vessels. The physical properties of ultrasound, which are acoustic energy waves with mechanical and thermal effects on insonated biological tissues, can be harnessed for diagnostic medical imaging as well as therapeutic purposes. The acoustic energy of ultrasound consists of mechanical longitudinal waves with pressure amplitudes that can be spatially directed and focused as a geometric beam with acoustic power and intensity, is measured in joules or watts per square centimeter (W/cm^2^). Ultrasound can be transmitted as intermittent pulses (pulsed-wave ultrasound) or continuously (continuous-wave ultrasound). During transmission and interaction with tissues, portions of the acoustic energy within the ultrasound beam are reflected, transmitted, and absorbed, depending on the acoustic impedance of the insonated tissues. The extent of these interactions is also influenced by the ultrasound instrumentation and technique, including operating mode, transducer design, application technique, and the total acoustic energy output.^3^^,^^4^

**Figure 1:**
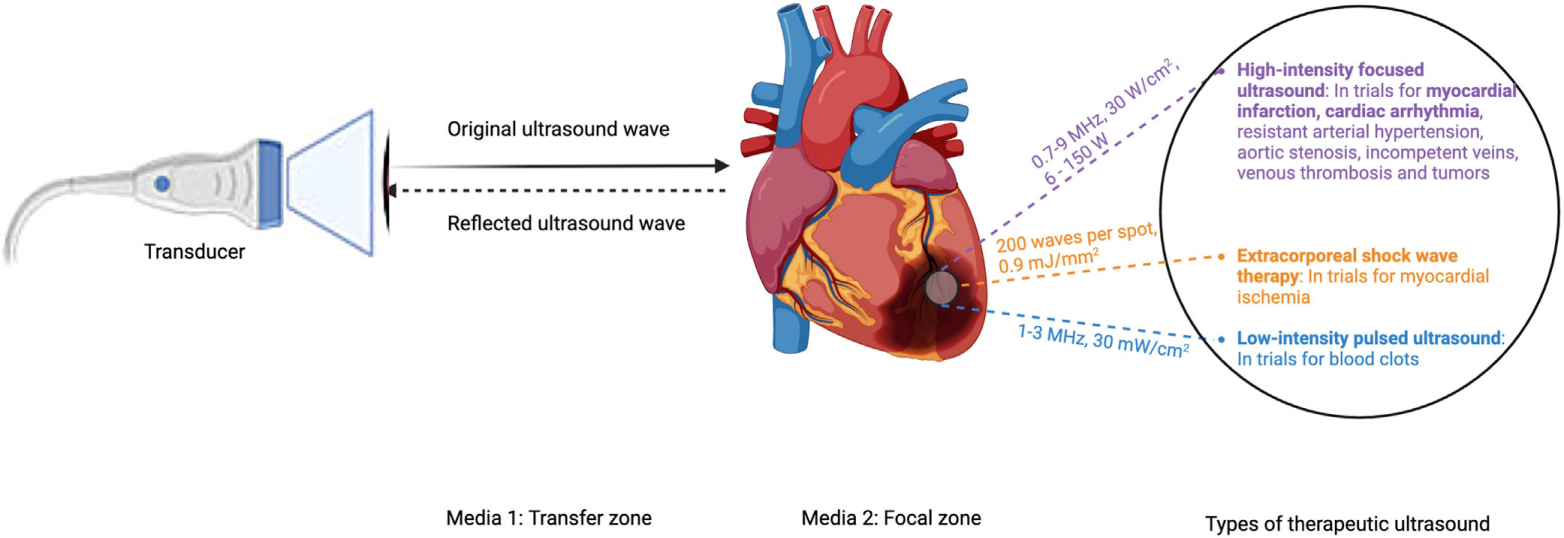
Cardiovascular ultrasound-based therapies. Created with BioRender.com (2023).

For several years, researchers have investigated the therapeutic use of ultrasound. More recently, there has been a surge of interest in therapeutic ultrasound for the treatment of CVDs. Advances in bioengineering and endovascular treatments have allowed for more accurate delivery of high-powered ultrasound, opening new avenues for the therapeutic use of ultrasound^5^, ^6^, ^7^, ^8^ (Figure 1). The therapeutic benefits of ultrasound are determined by its frequency, power, and whether it is targeted. This paper presents a contemporary overview of therapeutic applications of ultrasound in patients with CVDs.

## Ultrasound-Based Therapies: Mechanisms of Action in CVDs

Ultrasound-based therapies are commonly associated with physical therapy. In the realm of CVDs, ultrasound is seldom used therapeutically and is still under investigation. Its mechanism of action is further summarized in Tables 1 and 2, Figure 1, and below, based on the type of sound waves.

**Table 1:** Thermal vs. nonthermal effects of ultrasound therapy

Therapy	Type	Mechanism	Applications
High-intensity focused ultrasound (HIFU)	Thermal	High-energy focused ultrasound waves induce tissue coagulation and ablation, concentrating energy to elevate temperature precisely in targeted areas, sparing surrounding tissue.^3^	- Ablation of arrhythmogenic tissue in atrial fibrillation - Noninvasive renal denervation for resistant hypertension
Extracorporeal shock wave therapy (ESWT)	Nonthermal	Utilizes mechanical effects of ultrasound waves such as vibration and pressure to stimulate biological responses without significant heat generation.^6^^,^^9^	- Treatment of myocardial ischemia by breaking up arterial plaques - Refractory angina pectoris
Low-intensity pulsed ultrasound (LIPUS)	Nonthermal	Low-energy pulsed ultrasound waves activate cellular processes and promote microbubble cavitation, facilitating healing and perfusion enhancement.^10^	- Enhancing muscle perfusion - Stroke recovery - Cardiac dysfunction amelioration
Pulsed cavitational ultrasound therapy	Nonthermal	Ultrasound pulses create cavitation bubbles that mechanically disrupt tissues on a microscale for therapeutic benefits.^11^	- Noninvasive cardiac ablation - Enhancing drug delivery efficiency

**Table 2:** Invasive vs. noninvasive ultrasound therapy

Therapy	Type	Method	Applications	Specific considerations
Extracorporeal shock wave therapy (ESWT)	Noninvasive	Therapy applied externally without the need for surgical intervention.^6^^,^^9^	- Myocardial ischemia - Refractory angina pectoris	- Patient comfort - Repeat sessions may be required
Pulsed cavitational ultrasound therapy	Noninvasive	Uses external devices to deliver therapeutic ultrasound waves.^11^	- Aortic valve stenosis - Noninvasive cardiac ablation	- Focused ultrasound to specific areas without surgery
Low-intensity pulsed ultrasound (LIPUS)	Noninvasive	Ultrasound therapy is delivered through the skin using portable or fixed devices.^10^	- Stroke recovery - Cardiac dysfunction - Home or clinic-based therapy for chronic conditions	- Regular sessions needed for effect
High-intensity focused ultrasound (HIFU)	Invasive	Therapy requiring surgical exposure or insertion into the body.^3^	- Ablation of arrhythmogenic tissue in AFIB during surgeries	- Surgical risks involved - Requires anesthesia
HIFU (invasive approaches)	Invasive	Invasive applications during interventional procedures.^12^	- Renal denervation	- Direct tissue targeting - Used in resistant hypertension cases

Abbreviations: AFib, atrial fibrillation; HIFU, high-intensity focused ultrasound.

### High-Intensity Focused Ultrasound

High-intensity focused ultrasound (HIFU) is a noninvasive therapeutic technique that delivers high-powered, focused ultrasound energy to targeted tissues. In 1942, Lynn first introduced HIFU for focal tissue ablation with minimal/no effects on surrounding tissue.^13^ The ultrasound parameters of HIFU include continuous waves with generally low frequencies (frequency: 0.7-9 MHz) and high intensities (30 W/cm^2^ and acoustic power: 6-150 W).^3^^,^^6^^,^^14^, ^15^, ^16^, ^17^ Lower frequencies enable deeper penetration for transthoracic approaches with less tissue attenuation and low thermal index^18^, whereas higher frequencies (3.8-9 MHz) are suited for more superficial structures due to lower depth of penetration caused by greater attenuation.^14^, ^15^, ^16^, ^17^ Ultrasound at frequencies in the MHz range can be safe and effective for treating ischemic stroke, as reported in 57 small clinical trials and in a meta-analysis of 10 studies.^12^^,^^19^ The biological effects of HIFU are generated in situ based on the properties of tissues and mediated by thermal and/or mechanical effects.^20^ HIFU’s thermal effects can be harnessed in cardiovascular medicine to ablate and isolate focal tissues that trigger atrial fibrillation, as well as in noncardiac applications such as renal denervation and malignancies.^20^ Khokhlova et al. (2014) investigated HIFU for noninvasive cardiac ablation in a porcine model, demonstrating its potential for precise cardiovascular tissue interventions not often associated with ultrasound techniques.^21^ In the “boiling histotripsy” method, HIFU mechanically generates mm-sized boiling bubble that interact with the ultrasonic field to fractionate porcine tissue into subcellular debris without producing additional thermal effects.^21^ Pulsed cavitational ultrasound therapy (thrombotripsy) has also been investigated for the recanalization of proximal deep venous thrombosis in a swine model.^11^

### Extracorporeal Shock Wave Therapy (ESWT)

Extracorporeal shock wave therapy (ESWT) techniques employ high-pressure, high-energy, short duration pulses of sound called shock waves generated outside the body (extracorporeally) to deliver energy to tissues. ESWT produces a mechanical action in the tissue, resulting in rapid pressure changes, tissue deformation, and cellular mechano-transduction. As shockwaves propagate through body tissues, it is the properties of the shockwave, e.g., its pressure, focus, and frequency that determine the depth of penetration and interaction with the target tissue. Best practice ESWT clinical treatment parameters and protocols are generally categorized into three energy categories based on total energy dose and the energy per unit area or energy flux density using the following ranges: low (<0.08-10 mJ/mm2), medium (0.08-0.28 mJ/mm^2^), and high (>0.29-0.60 mJ/mm^2^). The ESWT protocols include exposure to separate pulses with high amplitude and predominant nonthermal biomechanical effects (frequency: individual shock waves; acoustic power: 0.9 mJ/mm^2^; and delivery protocol: 200 shock waves per spot, 40 to 60 spots per session, three sessions per week for 3 ​months).^6^^,^^7^

### Low-Intensity Pulsed Ultrasound (LIPUS)

Low-intensity pulsed ultrasound (LIPUS) is a therapeutic modality that harnesses low-power, low-intensity ultrasound (intensities typically around 30 mW/cm^2^, frequencies 1 to 3 MHz, pulse duration of 200 ​μs, and pulse repetition frequency of 1 kHz)^22^ to exert a nonthermal biological effects on tissues, including reduced inflammation, improved blood flow, and healing by direct stimulation of tissues or indirectly by harnessing the therapeutic potential of lipid-coated or drug-loaded microbubbles.^7^^,^^10^^,^^23^ Microbubbles are microscopic, highly reflective gas-filled bubbles encapsulated in a stabilizing protein, lipid, or polymer shell that oscillate and cavitate when exposed to the mechanical pressures from ultrasound insonation.

## Clinical Applications of Ultrasound-Based Therapy in CVDs

Cardiovascular ultrasound-based therapies are a fast-developing discipline with the potential to become useful adjuncts in the treatment of CVDs. Therapeutic ultrasound may be utilized alone, as in stroke (sonolysis) to cause clot lysis and then recanalization,^24^ or combined with tissue-type plasminogen activator treatment to increase its effects (sonothrombolysis), or in conjunction with microbubbles.^12^^,^^25^ The following discourse and Table 2 summarize various clinical applications of therapeutic ultrasound modalities based on the mode of delivery, which includes invasive and noninvasive techniques.

### Noninvasive Techniques

#### Sonothrombolysis

Sonothrombolysis holds significant promise due to its potential to eliminate or significantly reduce reliance on thrombolytic agents for clot dissolution. The primary mechanism of action of sonothrombolysis is mediated through acoustic radiation force, localized tissue displacement, and clot deformation.^26^ Animal studies showed that LIPUS could be beneficial in **stroke**,^27^ **ischemic heart disease,**^28^ and contractile dysfunction,^29^ likely through upregulation of vascular endothelial growth factor (VEGF) and endothelial nitric oxide synthase.^28^ The ultrasonic vibrations induce cells to release proteins and other chemicals that aid in healing and inflammation reduction. A study^30^ found that lower-frequency ultrasound is more effective at dissolving blood clots than higher-frequency ultrasound. This finding is important because it suggests that ultrasound-induced blood clot dissolution may be a viable alternative to thrombolytic drugs in certain settings.

##### Sonothrombolysis in Myocardial Infarction

**Figure 2:**
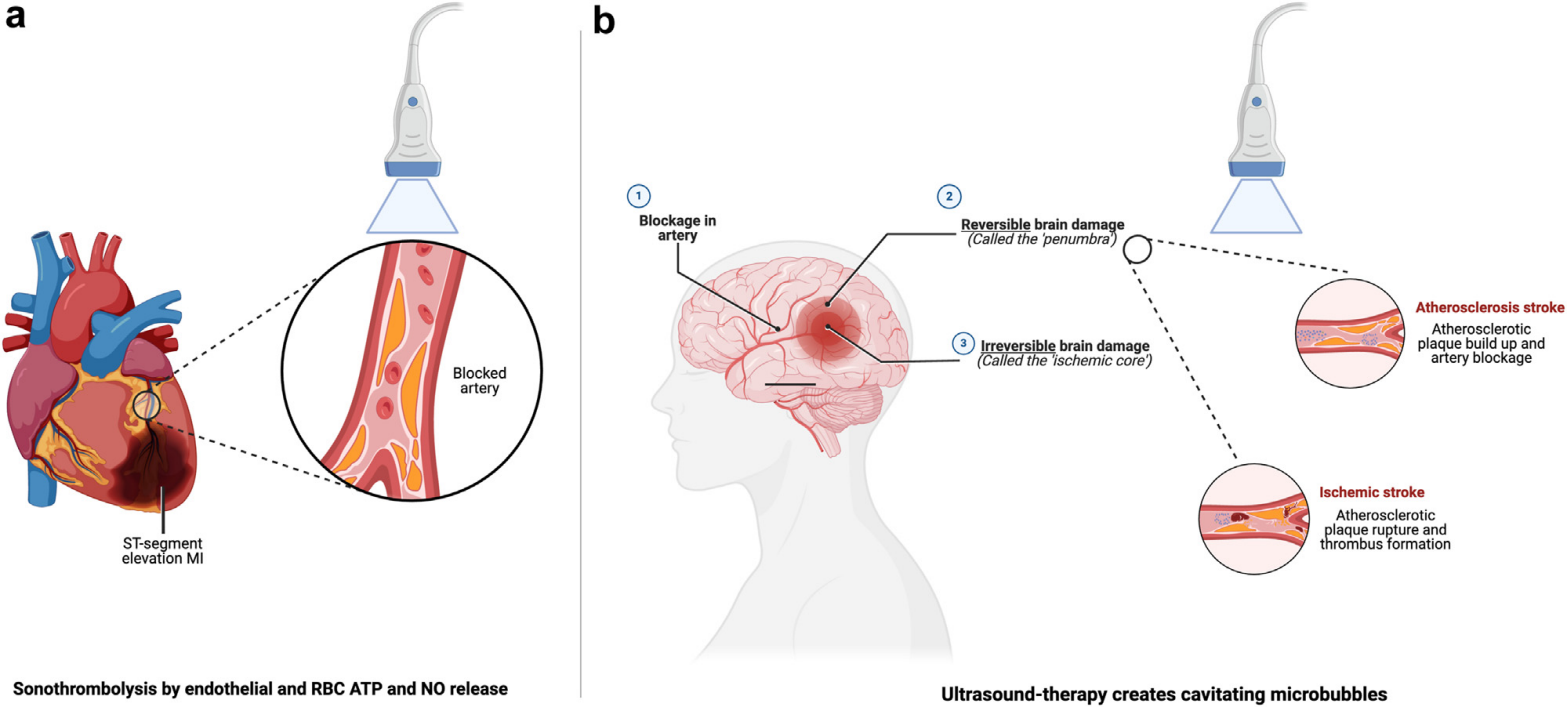
Sonothrombolysis in cardiovascular diseases. Adapted from “Common Types of Stroke,” “Reversible vs. Irreversible Brain Damage Following an Ischemic Stroke,” and “Myocardial Infarction (Heart Attack)” by BioRender.com (2024). References^32^, ^33^, ^34^, ^35^, ^36^, ^37^ Abbreviations: ATP, adenoside triphosphate; MI, myocardial infarction; NO, nitric oxide; RBC, red blood cell.

Sonothrombolysis is a therapeutic intervention that combines ultrasound techniques combined with thrombolytic therapy to enhance the treatment of myocardial infarction, particularly ST-segment-elevation myocardial infarction. It is conducted by a trained medical professional and lasts for approximately 20 ​minutes or until the patient reaches the hospital or interventional laboratory. The procedure involves the use of proprietary microbubble infusions with simultaneous transthoracic echocardiography. High mechanical index (MI) instrument settings (MI of approximately 1.0 to 1.2) with transmit frequency of 1.6 MHz and pulse duration less than 5 μs with imaging averaging 20 frames^31^ (Figure 2A). Typically, 30 to 60 impulses are administered, with 4 to 8 ​seconds between each high MI pulse to ensure sufficient replenishment of the microbubble contrast within the perfusion beds.

Preprimary percutaneous coronary intervention (pPCI) sonothrombolysis was shown to be an important factor in improving myocardial salvage in ST-segment-elevation myocardial infarction in the REDUCE Pilot trial.^38^ It improved myocardial salvage by an additional 17% compared to post-pPCI sonothrombolysis alone. This benefit was not affected by the duration of post-pPCI sonothrombolysis.^38^

##### Sonothombolysis in Cerebrovascular Stroke

After accumulating a substantial body of preclinical evidence regarding the potential efficacy of sonothrombolysis in addressing middle cerebral artery occlusions, numerous clinical trials have been conducted. These trials have employed transcranial Doppler ultrasound for treating middle cerebral artery occlusions, both with thrombolytic agents alone^39^, ^40^, ^41^ and in combination with microbubbles^32^^,^^33^^,^^42^ (Figure 2B).

## Extracorporeal Cardiac Shock Wave Therapy

Research has shown that a low level of extracorporeal cardiac shock wave therapy (ECSWT) can upregulate VEGF, a potent mitogen that triggers angiogenesis in human umbilical vascular endothelial cells to ameliorate myocardial ischemia and refractory angina pectoris.^3^^,^^43^, ^44^, ^45^, ^46^ Cardiac shockwave treatment includes three times a week, 200 shoots/spot at 0.09 mJ/mm^2^ for 45 spots in the ischemic area each time with an electromagnetic shockwave source (Storz Medical, Kreuzlingen, Switzerland).

While shockwave therapy uses sound waves, it differs from ultrasound in frequency and application. Therefore, it falls outside the strict definition of ultrasound but remains "ultrasound-adjacent." Free radical and epoxide production are theorized mechanisms of action.^3^ This may account for the immediate analgesic effects. Myocardial perfusion and function improvements may be the result of shockwave therapy-stimulated neo-angiogenesis.^3^ ECSWT is not yet indicated as a standard treatment in cases of **myocardial ischemia,** which is likely due to long duration of treatment, which requires transducer positioning through an intercostal space by a trained sonographer, despite potential upregulation of VEGF-Flt system.^47^ Interestingly, Kikuchi et al. (2010) conducted a double-blind, placebo-controlled study of ECSWT in individuals with severe angina pectoris.^43^ They found that ECSWT significantly improved symptoms and exercise tolerance compared to placebo, although a larger study is needed. Fukumoto et al.^44^ (2006) conducted a study of ECSWT in 9 patients with end-stage coronary artery disease. They found that ECSWT improved myocardial perfusion in the ischemic area as assessed by dipyridamole stress thallium scintigraphy, and effects persisted for 12 months. ECSWT may be a particularly good option for patients with severe coronary artery disease who are not candidates for revascularization procedures.

## Pulsed Cavitational Focused Ultrasound

The pulsed cavitational focused ultrasound is engineered to enhance the functionality of calcified aortic valves (AVs) through noninvasive ultrasound-based therapy. Its mechanism involves generating microscopic cavitation bubbles using short, high-pressure ultrasound pulses focused on calcified tissues, such as those seen in calcific AV stenosis. As these bubbles collapse, they create shock waves that mechanically soften the targeted calcified valve leaflets. In the study by Messas et al.^48^ (2021), the researchers evaluated the feasibility and performance of noninvasive ultrasound-based therapy in ten patients with severe symptomatic AV stenosis. In this study, patients who received the most ultrasound-based therapy time and energy demonstrated encouraging results. More recently, Messas et al. demonstrated improved AV function up to 6 months after noninvasive ultrasound-based therapy in 40 adult patients with severe symptomatic AV stenosis.^49^

## Hypertension

There is early and growing evidence for its important role of HIFU in the treatment of **resistant arterial hypertension** through renal denervation.^50^ A clinical study^50^ demonstrated that noninvasive renal denervation using HIFU resulted in a mean reduction in 24-hour ambulatory blood pressure of -13.1/-7.6 (standard deviation [SD] 9.5/8.5) mmHg, -14.9/-9.0 (SD 8.7/7.0) mmHg, and -11.4/-4.8 (SD 4.8/4.8) mmHg from baseline to 1, 3, and 6 months, respectively.

## Invasive Techniques

### Thrombosis and Embolism

The EkoSonic Endovascular System (EKOS) catheter is marketed (Boston Scientific) as a Food and Drug Administration (FDA)-approved catheter-based treatment that uses a combination of ultrasound waves and drugs to dissolve blood clots that cause pulmonary embolism or deep vein thrombosis. Its clinical utility was shown to be effective in the ULTIMA,^51^ SEATTLE II,^52^ OPTALYSE,^53^ and KNOCOUT^54^ trials. In patients with pulmonary embolism at intermediate risk, the ULTIMA study^51^ (n = 59) found ultrasound-assisted catheter-directed thrombolysis to be superior to anticoagulants alone up to 24 ​hours postprocedure, with no substantial bleeding risk. In patients with acute massive and submassive pulmonary embolism, SEATTLE II trial^52^ (n = 150) found ultrasound-facilitated, catheter-directed, low-dose fibrinolysis decreased right ventricular dilation, pulmonary pressure, anatomic thrombus burden, and minimized intracranial hemorrhage up to 72 ​hours postprocedure. OPTALYSE trial^53^ (n = 101) suggested that lower drug doses and shorter infusion times of ultrasound-facilitated catheter-directed thrombolysis were linked to improved right ventricular function and reduced clot burden, with low risk of bleeding up to 48 ​hours postprocedure. KNOCOUT trial^54^ (n = 1500) furthered the understanding of OPTALYSE protocol adoption and showed a reduction of the right ventricular to left ventricular diameters (RV/LV) ratio up to 14 days postprocedure. Future large-scale, multicenter clinical trials are needed to determine the efficacy and safety of such protocols in the long term.

Shockwave Medical has FDA-approved devices utilizing intravascular lithotripsy for managing severely calcific plaque lesions that use acoustic shockwaves in a balloon-based delivery system.^55^^,^^56^ LIPUS is also investigated for ultrasound-assisted thrombolysis of **acute pulmonary embolism**,^57^, ^58^, ^59^ right ventricular dysfunction, and heart failure with preserved left ventricular ejection fraction (in animal models^60^^,^^61^). Previous studies compared the effectiveness of ultrasound-assisted catheter-directed thrombolysis and conventional catheter-directed thrombolysis and concluded that both were equally effective for the treatment of acute pulmonary embolism.^58^^,^^59^ However, it is not recommended as a standard treatment for acute pulmonary embolism. There was no clear advantage of using one treatment over the other; nevertheless, the rate of moderate or severe bleeding was higher in ultrasound-assisted thrombolysis intervention.^58^ Similarly, a Dutch study^57^ concluded that ultrasound-assisted thrombolysis is associated with a high rate of major bleeding in patients with high-risk pulmonary embolism. Therefore, caution needs to be taken particularly in individuals at risk of bleeding.

### Arrhythmias

Ultrasound-based therapy benefits still outweigh the harms in **cardiac arrhythmias** (HIFU, moderate quality evidence^16^^,^^62^, ^63^, ^64^). If medical therapy is not effective in treating cardiac arrhythmia, an ablation can be performed with the goal of thermally removing the arrhythmogenic tissue. This can be used to ablate circuits to stop arrhythmia re-entrant circuits or to inhibit areas of focus. HIFU using the epicardial method has been applied in cardiac surgeries. Reyes et al.^62^ (2016) reported the results of the National Spanish Registry of HIFU ablation for atrial fibrillation. The overall efficacy of atrial fibrilliation ablation with the HIFU Epicor system was 63.9 and 45.9% at 24- and 36-month follow-up. The outcomes of a multicenter study of surgical epicardial HIFU ablation for atrial fibrillation were reported by Ninet et al. (2005).^63^ Eighty percent of individuals with persistent atrial fibrillation associated with long-standing structural heart disease were successfully cured with epicardial, off-pump, beating-heart ablation using HIFU. The findings of a research on epicardial HIFU for the surgical management of atrial fibrillation were published in 2009 by Mitnovetski et al.^64^ At the time of publication, 10/13 (77%) of the individuals had sinus rhythm; one needed a permanent pacemaker implantation; one was in atrial fibrillation; and another was in atrial flutter. Schopka et al. (2010) reported the results of a prospective clinical study of epicardial HIFU ablation for atrial fibrillation.^16^ Although 62% of patients at 6 months had sinus rhythms, overall conversion rates were lower than in earlier publications. Moreover, preclinical research indicates that an endocardial strategy for treating mid-myocardial or epicardial ventricular arrhythmias may be effective with HIFU catheter ablation.^8^

### Hypertension

There is early and growing evidence for its important role of HIFU in the treatment of **resistant arterial hypertension** through renal denervation.^65^ The sympathetic nervous system in the kidneys plays a crucial role in regulating and contributing to the development of hypertension. Consequently, ultrasound-based renal denervation holds the potential to reduce blood pressure. This technique is usually performed with an endovascular approach. The recent RADIANCE II randomized controlled trial demonstrated efficacy and safety of ultrasound renal denervation in reducing blood pressure in patients with systemic hypertension without postoperative major adverse events.^66^ This resulted in FDA approval of PARADISE ultrasound denervation for treatment of hypertension in 2023.

### Ischemic Heart Failure

Recently, an Austrian trial indicated that direct cardiac shockwave therapy, combined with coronary bypass surgery, improves left ventricular ejection fraction (LVEF) and physical capacity in patients with ischemic heart failure. Larger trials are needed to confirm if direct cardiac shockwave therapy improves myocardial function, as suggested here.^67^

### Miscellaneous

The role of ultrasound-based therapy is also being explored for managing **incompetent veins** (noninvasively and thermogenically closing a malfunctioning vein permanently^68^^,^^69^), **hypertrophic cardiomyopathy,** and **external cardiac pacing** through cardiac stimulation (preclinical data^70^, ^71^, ^72^). A case report^68^ demonstrated that extracorporeal HIFU treatment of an incompetent perforating vein in a 95-year-old man with active venous ulcers was successful. Three months after HIFU, reflux was eliminated, and the two ulcers that were initially active had healed. Whiteley^69^ also suggested that HIFU is a promising treatment for varicose veins and venous leg ulcers. The effectiveness of therapeutic ultrasonography, at either high or low frequency, in accelerating the healing of venous leg ulcers is unknown.^73^ There is some limited evidence of enhanced healing when using therapeutic ultrasound^73^; however, further research is needed to evaluate the long-term efficacy and safety of HIFU in these patients.

## General Considerations and Implications

Ultrasound-based therapies represent emerging modalities in the management of complex cardiovascular conditions. As these innovative approaches gain traction, several key considerations must be thoroughly addressed to ensure their effective integration into clinical practice.

### Safety and Efficacy

The foremost concern with the introduction of any new therapeutic technology is its safety and efficacy. HIFU procedures require vigilance due to possible thermal injury, potentially causing unintended tissue damage if not properly controlled and monitored. Cooling and temperature monitoring are crucial for mitigating this risk. ECWT and ultrasound-facilitated thrombolysis can induce mechanical stress, leading to vascular injury if not carefully administered. Careful selection of acoustic parameters and cavitation monitoring are essential for safety.^6^^,^^74^^,^^75^ Ultrasound-based therapies must be validated through rigorous, large-scale, randomized clinical trials that not only assess the immediate therapeutic outcomes but also long-term effects on patient health and disease progression. For cardiologists, involvement goes beyond clinical application; it extends to active participation in research and critical evaluation of both positive outcomes and potential adverse effects. Ensuring that these technologies do more good than harm is paramount, and this can only be achieved with comprehensive data that supports their use in specific cardiovascular scenarios.

### Integration Into Practice

Integrating therapeutic ultrasound into routine cardiology practice requires a multifaceted approach. Firstly, practitioners need detailed training to understand the nuances of ultrasound technologies, including the technical aspects of equipment and the physiological implications of their use. Secondly, clear clinical guidelines must be developed to delineate indications for use, procedural protocols, and contraindications. These guidelines should be evidence-based to ensure standardized care across different health care settings. Furthermore, effective integration relies on interdisciplinary collaboration among cardiologists, sonographers, bioengineers, and other health care professionals to fine-tune the application of these technologies and tailor them to individual patient needs.

### Patient Selection

The success of ultrasound-based therapy greatly depends on selecting the right patients. Factors such as the specific cardiac anatomy, the nature and stage of the CVDs, and the overall health condition of the patient play critical roles in determining the appropriateness of ultrasound-based therapies. For instance, patients with complex cardiac geometries or those with certain types of tissue characteristics may benefit more from HIFU or ESWT, whereas others may be better suited for LIPUS based on the desired therapeutic outcomes and risk profiles. Precision in patient selection ensures that the applied technology provides maximum benefit and minimizes risks, thus leveraging the full potential of therapeutic ultrasound in personalized medicine.

### Cost-Effectiveness and Accessibility

Another significant consideration is the cost-effectiveness of these ultrasound technologies compared to traditional treatments. Health economics should be considered, assessing not only the direct costs associated with the technology itself but also the potential reduction in overall health care expenses through improved management of CVDs. Additionally, the accessibility of these technologies in different health care settings, including under-resourced environments, must be evaluated to ensure equitable health care delivery.

### Future Perspectives

Looking forward, the continued advancement in ultrasound technology promises to expand the boundaries of what is currently achievable in cardiovascular therapies. Innovations in ultrasound technology could lead to more precise interventions, reduced invasiveness, and enhanced patient outcomes. Embracing these technologies also means preparing for a future where cardiology practice may increasingly rely on advanced imaging and therapeutic modalities as integral components of cardiovascular care.

## Conclusion

The efficacy of therapeutic ultrasound in ST elevation myocardial infarction and cardiac arrhythmia has been demonstrated through clinical trials when used alongside standard therapy. However, its potential for treating tumors, resistant arterial hypertension, venous leg ulcers, and acute pulmonary embolism is not yet fully established. There are concerns regarding potential side effects, such as excessive bleeding in individuals at risk. The ongoing challenge lies in determining the optimal intensity, frequency, and duration of ultrasound exposure for patients with CVDs.

## CRediT Authorship Contributions

Amit Bansal drafted the original manuscript and submitted it. Later, additional authors were invited to contribute. Bernard Bulwer and Ricarda von Krüchten then undertook extensive rewriting of the manuscript. Jagkirat Singh provided a review of the revised version. Rajan Rehan created the tables and reviewed the overall work. Finally, Ata Doost also offered extensive feedback and contributed to the generation of the second figure.

## Funding

Dr Amit Bansal received funding from the 10.13039/501100005036University of Bergen, Norway, The National 10.13039/100022395Graduate School in Infection Biology and Antimicrobials (or IBA), and Pasteur legatet & Thjøtta’s legat, 10.13039/501100005366University of Oslo, Norway [101563].

## Acknowledgments

We pay tribute to health care workers for their altruistic translational work.

## Disclosure Statement

The authors report no conflict of interest.

## References

[1] Vaduganathan M., Mensah G.A., Turco J.V., Fuster V., Roth G.A. (2022). The global burden of cardiovascular diseases and risk: a Compass for future health. J Am Coll Cardiol.

[2] Mensah G.A., Roth G.A., Fuster V. (2019). The global burden of cardiovascular diseases and risk factors: 2020 and beyond. J Am Coll Cardiol.

[3] Nesser H.J., Karia D.H., Tkalec W., Pandian N.G. (2002). Therapeutic ultrasound in cardiology. Herz.

[4] Nyborg W.L., Carson P.L., Dunn F. (1983). Biological Effects of Ultrasound: Mechanisms and Clinical Implications. National Council on Radiation Protection and Measurements.

[5] Liu T., Shi J., Fu Y. (2023). New trends in non-pharmacological approaches for cardiovascular disease: therapeutic ultrasound. Trends Cardiovasc Med.

[6] Nazer B., Gerstenfeld E.P., Hata A., Crum L.A., Matula T.J. (2014). Cardiovascular applications of therapeutic ultrasound. J Interv Card Electrophysiol.

[7] Nazer B., Ghahghaie F., Kashima R. (2015). Therapeutic ultrasound promotes Reperfusion and angiogenesis in a Rat model of Peripheral arterial disease. Circ J.

[8] Nazer B., Giraud D., Zhao Y. (2021). High-intensity ultrasound catheter ablation achieves deep mid-myocardial lesions in vivo. Heart Rhythm.

[9] Ling Z.Y., Shu S.Y., Zhong S.G. (2013). Ultrasound targeted microbubble destruction promotes angiogenesis and heart function by inducing myocardial microenvironment change. Ultrasound Med Biol.

[10] Graham S.M., Carlisle R., Choi J.J. (2014). Inertial cavitation to non-invasively trigger and monitor intratumoral release of drug from intravenously delivered liposomes. J Control Release.

[11] Goudot G., Khider L., Del Giudice C. (2020). Non-invasive recanalization of deep venous thrombosis by high frequency ultrasound in a swine model with follow-up. J Thromb Haemost.

[12] Leinenga G., Langton C., Nisbet R., Götz J. (2016). Ultrasound treatment of neurological diseases — current and emerging applications. Nat Rev Neurol.

[13] Lynn J.G., Zwemer R.L., Chick A.J., Miller A.E. (1942). A new method for the generation and use of focused ultrasound in experimental biology. J Gen Physiol.

[14] Takei Y., Muratore R., Kalisz A. (2012). In vitro atrial septal ablation using high-intensity focused ultrasound. J Am Soc Echocardiogr.

[15] Haqqani H.M., Tschabrunn C.M., Tzou W.S. (2011). Isolated septal substrate for ventricular tachycardia in nonischemic dilated cardiomyopathy: incidence, characterization, and implications. Heart Rhythm.

[16] Schopka S., Schmid C., Keyser A. (2010). Ablation of atrial fibrillation with the Epicor system: a prospective observational trial to evaluate safety and efficacy and predictors of success. J Cardiothorac Surg.

[17] Nakagawa H., Antz M., Wong T. (2007). Initial experience using a forward directed, high-intensity focused ultrasound balloon catheter for pulmonary vein antrum isolation in patients with atrial fibrillation. J Cardiovasc Electrophysiol.

[18] Suchkova FNS V., Carstensen E.L., Dalecki D., Child S., Francis C.W. (1998). Enhancement of fibrinolysis with 40-kHz ultrasound. Circulation.

[19] Saqqur M., Tsivgoulis G., Nicoli F. (2014). The role of sonolysis and sonothrombolysis in acute ischemic stroke: a Systematic review and meta-analysis of randomized controlled trials and case-Control studies. J Neuroimaging.

[20] Bachu V.S., Kedda J., Suk I., Green J.J., Tyler B. (2021). High-intensity focused ultrasound: a review of mechanisms and clinical applications. Ann Biomed Eng.

[21] Khokhlova T.D., Wang Y.-N., Simon J.C. (2014). Ultrasound-guided tissue fractionation by high intensity focused ultrasound in an in vivo porcine liver model. Proc Natl Acad Sci USA.

[22] Khanna A., Nelmes R.T., Gougoulias N., Maffulli N., Gray J. (2009). The effects of LIPUS on soft-tissue healing: a review of literature. Br Med Bull.

[23] Li J., Zhang Q., Ren C. (2018). Low-intensity pulsed ultrasound Prevents the Oxidative stress induced endothelial-Mesenchymal Transition in human aortic endothelial cells. Cell Physiol Biochem.

[24] Barreto A.D., Sharma V.K., Lao A.Y. (2009). Safety and dose-Escalation study design of transcranial ultrasound in clinical sonolysis for acute ischemic stroke: the TUCSON trial. Int J Stroke.

[25] Barreto A.D., Alexandrov A.V., Shen L. (2013). CLOTBUST-Hands Free: pilot safety study of a novel operator-independent ultrasound device in patients with acute ischemic stroke. Stroke.

[26] Jones G., Hunter F., Hancock H.A. (2010). In vitro investigations into enhancement of tPA bioavailability in whole blood clots using pulsed-high intensity focused ultrasound exposures. IEEE Trans Biomed Eng.

[27] Ichijo S., Shindo T., Eguchi K. (2021). Low-intensity pulsed ultrasound therapy promotes recovery from stroke by enhancing angio-neurogenesis in mice in vivo. Sci Rep.

[28] Hanawa K., Ito K., Aizawa K. (2014). Low-intensity pulsed ultrasound induces angiogenesis and ameliorates left ventricular dysfunction in a porcine model of chronic myocardial ischemia. PLOS ONE.

[29] Ogata T., Ito K., Shindo T. (2017). Low-intensity pulsed ultrasound enhances angiogenesis and ameliorates contractile dysfunction of pressure-overloaded heart in mice. PLoS One.

[30] Nedelmann M., Brandt C., Schneider F. (2005). Ultrasound-induced blood clot dissolution without a thrombolytic drug is more effective with lower frequencies. Cerebrovasc Dis.

[31] El Kadi S., Porter T.R., van Rossum A.C., Kamp O. (2021). Sonothrombolysis in the ambulance for ST-elevation myocardial infarction: rationale and protocol. Neth Heart J.

[32] Molina C.A., Ribo M., Rubiera M. (2006). Microbubble administration accelerates clot lysis during continuous 2-MHz ultrasound monitoring in stroke patients treated with intravenous tissue plasminogen activator. Stroke.

[33] Ribo M., Molina C.A., Alvarez B., Rubiera M., Alvarez-Sabin J., Matas M. (2010). Intra-arterial administration of microbubbles and continuous 2-MHz ultrasound insonation to enhance intra-arterial thrombolysis. J Neuroimaging.

[34] Kuriakose D., Xiao Z. (2020). Pathophysiology and treatment of stroke: Present Status and future Perspectives. Int J Mol Sci.

[35] BioRender Common types of stroke. https://app.biorender.com/biorender-templates/figures/all/t-63a4b78a4091411743b1f6b5-common-types-of-stroke.

[36] BioRender Reversible vs. Irreversible brain damage following an ischemic stroke. https://app.biorender.com/biorender-templates/figures/all/t-5ea9fdcb10cf9500aae49fc7-reversible-vs-irreversible-brain-damage-following-an-ischemi.

[37] (2022). BioRender. https://app.biorender.com/biorender-templates/figures/all/t-62e70371f2dd732bb68c3cd1-myocardial-infarction-heart-attack.

[38] Nirthanakumaran D., Jeyaprakash P., Pathan F. (2023). Sonothrombolysis delivered before percutaneous coronary intervention improves myocardial salvage in ST elevation myocardial infarction. Eur Heart J.

[39] Daffertshofer M., Gass A., Ringleb P. (2005). Transcranial low-frequency ultrasound-mediated thrombolysis in brain ischemia: increased risk of hemorrhage with combined ultrasound and tissue plasminogen activator: results of a phase II clinical trial. Stroke.

[40] Alexandrov A.V., Molina C.A., Grotta J.C. (2004). Ultrasound-enhanced systemic thrombolysis for acute ischemic stroke. N Engl J Med.

[41] Alexandrov A.V., Tsivgoulis G., Kohrmann M. (2019). Endovascular equipoise shift in a phase III randomized clinical trial of sonothrombolysis for acute ischemic stroke. Ther Adv Neurol Disord.

[42] Perren F., Loulidi J., Poglia D., Landis T., Sztajzel R. (2008). Microbubble potentiated transcranial duplex ultrasound enhances IV thrombolysis in acute stroke. J Thromb Thrombolysis.

[43] Kikuchi Y., Ito K., Ito Y. (2010). Double-blind and placebo-controlled study of the effectiveness and safety of extracorporeal cardiac shock wave therapy for severe angina pectoris. Circ J.

[44] Fukumoto Y., Ito A., Uwatoku T. (2006). Extracorporeal cardiac shock wave therapy ameliorates myocardial ischemia in patients with severe coronary artery disease. Coron Artery Dis.

[45] Khattab A.A., Brodersen B., Schuermann-Kuchenbrandt D. (2007). Extracorporeal cardiac shock wave therapy: first experience in the everyday practice for treatment of chronic refractory angina pectoris. Int J Cardiol.

[46] Prinz C., Lindner O., Bitter T. (2009). Extracorporeal cardiac shock wave therapy ameliorates clinical symptoms and improves regional myocardial blood flow in a patient with severe coronary artery disease and refractory angina. Case Rep Med.

[47] Nishida T., Shimokawa H., Oi K. (2004). Extracorporeal cardiac shock wave therapy markedly ameliorates ischemia-induced myocardial dysfunction in pigs in vivo. Circulation.

[48] Messas E., A I.J., Goudot G. (2021). Feasibility and performance of Noninvasive ultrasound therapy in patients with severe symptomatic aortic valve stenosis: a first-in-human study. Circulation.

[49] Messas E., Ijsselmuiden A., Trifunovic-Zamaklar D. (2023). Treatment of severe symptomatic aortic valve stenosis using non-invasive ultrasound therapy: a cohort study. Lancet.

[50] Rong S., Zhu H., Liu D. (2015). Noninvasive renal denervation for resistant hypertension using high-intensity focused ultrasound. Hypertension.

[51] Kucher N., Boekstegers P., Muller O.J. (2014). Randomized, controlled trial of ultrasound-assisted catheter-directed thrombolysis for acute intermediate-risk pulmonary embolism. Circulation.

[52] Piazza G., Hohlfelder B., Jaff M.R. (2015). A prospective, Single-Arm, multicenter trial of ultrasound-facilitated, catheter-directed, low-dose fibrinolysis for acute massive and submassive pulmonary embolism: the SEATTLE II study. JACC Cardiovasc Interv.

[53] Tapson V.F., Sterling K., Jones N. (2018). A randomized trial of the Optimum duration of acoustic pulse thrombolysis procedure in acute intermediate-risk pulmonary embolism: the OPTALYSE PE trial. JACC Cardiovasc Interv.

[54] Sterling K. (2024). An International pulmonary embolism Registry using EKOS (KNOCOUT PE). ClinicalTrials.gov ID NCT03426124. NCT03426124.

[55] Kereiakes D.J., Virmani R., Hokama J.Y. (2021). Principles of intravascular lithotripsy for calcific plaque Modification. JACC Cardiovasc Interv.

[56] Shockwave medical. https://shockwavemedical.com/clinicians/usa/coronary/.

[57] de Winter M.A., Hart E.A., van den Heuvel D.A.F. (2019). Local ultrasound-facilitated thrombolysis in high-risk pulmonary embolism: first Dutch experience. Cardiovasc Intervent Radiol.

[58] Rao G., Xu H., Wang J.J. (2019). Ultrasound-assisted versus conventional catheter-directed thrombolysis for acute pulmonary embolism: a multicenter comparison of patient-centered outcomes. Vasc Med.

[59] Liang N.L., Avgerinos E.D., Marone L.K., Singh M.J., Makaroun M.S., Chaer R.A. (2016). Comparative outcomes of ultrasound-assisted thrombolysis and standard catheter-directed thrombolysis in the treatment of acute pulmonary embolism. Vasc Endovascular Surg.

[60] Nakata T., Shindo T., Ito K. (2023). Beneficial effects of low-intensity pulsed ultrasound therapy on right ventricular dysfunction in animal models. JACC Basic Transl Sci.

[61] Monma Y., Shindo T., Eguchi K. (2021). Low-intensity pulsed ultrasound ameliorates cardiac diastolic dysfunction in mice: a possible novel therapy for heart failure with preserved left ventricular ejection fraction. Cardiovasc Res.

[62] Reyes G., Ruyra X., Valderrama F. (2016). High intensity focused ultrasound ablation for atrial fibrillation: results from the National Spanish Registry. Minerva Cardioangiol.

[63] Ninet J., Roques X., Seitelberger R. (2005). Surgical ablation of atrial fibrillation with off-pump, epicardial, high-intensity focused ultrasound: results of a multicenter trial. J Thorac Cardiovasc Surg.

[64] Mitnovetski S., Almeida A.A., Goldstein J., Pick A.W., Smith J.A. (2009). Epicardial high-intensity focused ultrasound cardiac ablation for surgical treatment of atrial fibrillation. Heart Lung Circ.

[65] Fengler K., Rommel K.P., Blazek S. (2019). A three-Arm randomized trial of different renal denervation devices and techniques in patients with resistant hypertension (RADIOSOUND-HTN). Circulation.

[66] Azizi M., Saxena M., Wang Y. (2023). Endovascular ultrasound renal denervation to Treat hypertension: the RADIANCE II randomized clinical trial. JAMA.

[67] Holfeld J, Nägele F, Pölzl L (2024). Cardiac shockwave therapy in addition to coronary bypass surgery improves myocardial function in ischaemic heart failure: the CAST-HF trial. Eur Heart J.

[68] Obermayer A., Aubry J.F., Barnat N. (2021). Extracorporeal treatment with high intensity focused ultrasound of an incompetent perforating vein in a patient with active venous ulcers. EJVES Vasc Forum.

[69] Whiteley M.S. (2020). High intensity focused ultrasound (HIFU) for the treatment of varicose veins and venous leg ulcers - a new non-invasive procedure and a potentially disruptive technology. Curr Med Res Opin.

[70] Miller D.L., Lu X., Dou C. (2018). Ultrasonic cavitation-Enabled treatment for therapy of hypertrophic cardiomyopathy: Proof of Principle. Ultrasound Med Biol.

[71] Livneh A., Kimmel E., Kohut A.R., Adam D. (2014). Extracorporeal acute cardiac pacing by high intensity focused ultrasound. Prog Biophys Mol Biol.

[72] Kohut A.R., Vecchio C., Adam D., Lewin P.A. (2016). The potential of ultrasound in cardiac pacing and rhythm modulation. Expert Rev Med Devices.

[73] Cullum N., Al-Kurdi D., Bell-Syer S.E.M. (2017). Therapeutic ultrasound for venous leg ulcers. Cochrane Database Syst Rev.

[74] Miller D.L., Smith N.B., Bailey M.R. (2012). Overview of therapeutic ultrasound applications and safety considerations. J Ultrasound Med.

[75] Ditac G., Bessière F., Lafon C. (2023). Therapeutic ultrasound applications in cardiovascular diseases: a review. IRBM.

